# Subspace Clustering of Physiological Data From Acute Traumatic Brain Injury Patients: Retrospective Analysis Based on the PROTECT III Trial

**DOI:** 10.2196/24698

**Published:** 2021-02-02

**Authors:** Sina Ehsani, Chandan K Reddy, Brandon Foreman, Jonathan Ratcliff, Vignesh Subbian

**Affiliations:** 1 Department of Systems and Industrial Engineering College of Engineering The University of Arizona Tucson, AZ United States; 2 Department of Computer Science Virginia Polytechnic Institute and State University Arlington, VA United States; 3 Department of Neurology & Rehabilitation Medicine University of Cincinnati Cincinnati, OH United States; 4 Department of Emergency Medicine Emory University School of Medicine Emory University Atlanta, GA United States; 5 Department of Biomedical Engineering College of Engineering, The University of Arizona Tucson, AZ United States

**Keywords:** cluster analysis, unsupervised machine learning, traumatic brain injury

## Abstract

**Background:**

With advances in digital health technologies and proliferation of biomedical data in recent years, applications of machine learning in health care and medicine have gained considerable attention. While inpatient settings are equipped to generate rich clinical data from patients, there is a dearth of actionable information that can be used for pursuing secondary research for specific clinical conditions.

**Objective:**

This study focused on applying unsupervised machine learning techniques for traumatic brain injury (TBI), which is the leading cause of death and disability among children and adults aged less than 44 years. Specifically, we present a case study to demonstrate the feasibility and applicability of subspace clustering techniques for extracting patterns from data collected from TBI patients.

**Methods:**

Data for this study were obtained from the Progesterone for Traumatic Brain Injury, Experimental Clinical Treatment–Phase III (PROTECT III) trial, which included a cohort of 882 TBI patients. We applied subspace-clustering methods (density-based, cell-based, and clustering-oriented methods) to this data set and compared the performance of the different clustering methods.

**Results:**

The analyses showed the following three clusters of laboratory physiological data: (1) international normalized ratio (INR), (2) INR, chloride, and creatinine, and (3) hemoglobin and hematocrit. While all subclustering algorithms had a reasonable accuracy in classifying patients by mortality status, the density-based algorithm had a higher F1 score and coverage.

**Conclusions:**

Clustering approaches serve as an important step for phenotype definition and validation in clinical domains such as TBI, where patient and injury heterogeneity are among the major reasons for failure of clinical trials. The results from this study provide a foundation to develop scalable clustering algorithms for further research and validation.

## Introduction

Traumatic brain injury (TBI) is broadly defined as disruption in normal brain function or other evidence of brain pathology as a result of mechanical force directed at the head or a rapid acceleration/deceleration event. TBI is the most common cause of death and disability in children and adults aged less than 44 years [1]. However, there has been little change in TBI-related deaths despite advancements in care delivery [2]. Additionally, a major challenge to both TBI-related clinical research and acute care is reliably identifying candidates for targeted interventions [3]. While there have been substantial advances in technological and computational approaches to TBI phenotyping [4-6], there is still a dearth of actionable information that can be used for pursing secondary clinical research in this domain.

Existing approaches to stratification of patients based on clinical presentation does not adequately address the heterogenous nature of TBI, whereas data mining and machine learning techniques have shown promise in identifying subgroups [5], predicting outcomes [7], and prognosticating among TBI patients [8]. In particular, clustering-based techniques serve as an important step for phenotype definition and have the potential to uncover previously unrecognized relationships between various physiologic variables [9]. For example, in other clinical domains, traditional cluster analyses have been helpful in identifying unique subgroups of patients. These studies include application of k-means cluster analysis for identifying distinct phenotypes of asthma patients [10], as well as using hierarchical clustering to identify both new and known relationships between physiologic variables collected from critically ill patients [9]. In this study, we applied *subspace clustering* (or subclustering) methods on physiologic data collected from TBI patients and compared the performance of different subspace clustering methods (density-based, cell-based, and clustering-oriented methods). The rationale for applying subspace clustering over traditional clustering methods (eg, k-means) is the ability to account for the multiple low-dimensional subspace structure of higher dimensional data [11]. In terms of critical illnesses, such as acute TBI, we hypothesize that the complex latent relationships between various physiologic variables are better represented in subspaces and thus better captured by subclustering methods than traditional methods that are often limited to spatial proximity of data points in individual clusters.

## Methods

### Data Source

Data for this study were obtained from the Progesterone for Traumatic Brain Injury, Experimental Clinical Treatment–Phase III (PROTECT III) study. The PROTECT III trial included a cohort of 882 TBI patients [12], who were originally recruited for a randomized clinical trial to study the effect of progesterone on patients with acute TBI. Patients were randomly assigned to a treatment group that received progesterone within 4 hours of injury or placebo. While the PROTECT III clinical trial showed that there was no difference in patients between the two study groups, the longitudinal data from the trial were made available for secondary analyses and continued research.

This data set included patient demographics, baseline assessment data, 6-month outcome data, including the Glasgow Outcome Scale Extended scale, and mortality status. The temporal data in this study included laboratory test results for the first 7 days of stay. Other clinical and radiologic data were not included in this analysis. Deidentified data were obtained in collaboration with the PROTECT III investigators and are now available through the Federal Interagency Traumatic Brain Injury Research informatics system. The inclusion criteria for this analysis were as follows: (1) subjects were alive for at least 3 days, (2) subjects were not excluded from the parent study, and (3) their baseline laboratory results were available. The 3-day criterion is used because subjects who do not survive for at least 3 days or 72 hours have likely experienced devastating brain injury or other forms of severe trauma, which often require aggressive interventions [13]. Additionally, the first 72 hours of observation is the time interval used for determining the preliminary effect of the injury and is thus recommended for valid prognostication [14,15].

### Subspace Clustering

Subspace or projective clustering is a clustering method that emphasizes on clustering in subspaces of high-dimensional spaces, that is, it tries to find clusters in smaller subspaces and builds up to form larger clusters by using overlapping subspaces [16]. Subspace clustering can be classified into the following three main categories: density-based approaches, cell-based approaches, and clustering-oriented approaches. Density-based approaches define subspaces in dense areas [17]. In cell-based approaches, subspaces are formed by predefining the width of grid cells and the number of objects within each cell [18]. Clustering-oriented approaches define properties of the entire set of clusters, as opposed to definition of the cluster itself, and then assign objects to the cluster with the most relevant properties [19].

#### Density-Based Approach

One of the commonly used clustering algorithms is density-based spatial clustering of applications with noise (DBSCAN) [20]. The key idea of DBSCAN is that after detecting a cluster using density-based grids, it looks at the neighborhood of each cluster point in a defined radius; any point that exists in this radius is contributed to the cluster.

Every cluster *C* in a subspace projection is defined by a set of objects *O*, that is a subset of database *DB* and a set of relevant dimensions *S* out of the set of all dimensions *D*.




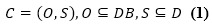




A clustering result *R* is a set of clusters *k* found in the respective subspace projections as follows:




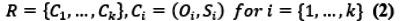




A density-based subspace cluster (O, S) in a two-dimensional space is defined with respect to parameters *minPoints* and *ε*–*neighborhood N_ε_* (*p*) = {*q* ∈ *DB* |*dist^S^* (*p, q*) ≤ *ε*}, where *dist^S^* represents a distance function constrained to the dimensions *S*, as follows [20]:

(1) *ε*–*neighborhood* of a point: Let p and q be two points of the sample, and the distance equation between these two points is defined by *dist* (*p*, *q*). The distance could be defined with Manhattan distance, Euclidean distance, or other different distance methods. The *ε*–*neighborhood* of a point is defined as follows:




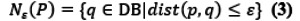




(2) *Directly density reachable:* A point p is directly density reachable from a point q with respect to *ε* and *MinPts* if




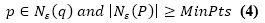




(3) *Density reachable:* A point p is density reachable from a point q with respect to *ε* and *MinPts* if all the points in a chain of points (including q and p) are directly density reachable from each another.

(4) *Density connected:* A point p is density connected to a point q if only there is point o, which both p and q are density reachable from.

(5) *Noise:* The sets of points in database DB that are not assigned to any cluster are called noise.

To find a cluster, the DBSCAN algorithm starts with a random point p and finds all density reachable points with respect to *ε* and *MinPts*. DBSCAN also merges two clusters together if the distance between two sets of points is defined as follows:


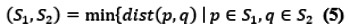


Density-connected subspace clustering (SUBCLU) is a greedy algorithm built on an adaption of the DBSCAN algorithm for high-dimensional data. It computes all density-connected sets hidden in subspaces of high-dimensional data. Studies have shown that SUBCLU can outperform other subspace clustering methods based on different measures [18,20,21]. SUBCLU is capable of detecting arbitrarily shaped clusters using the DBSCAN algorithm in subspaces. To use DBSCAN in each subspace, let DB be a d-dimensional feature vectors data set with n objects *DB* ⊆ *R^d^*. Let *A* = {*a_1_*, *a_2_*,…, *a_d_*} be the set of all attributes *a* of *DB*. Any subset *S* ⊆ *A* is called a subspace. The projection of an object *o* into a subspace *S* is denoted by *π_s_* (*o*), and the distance function is denoted by d*ist*. For instance, the *ε*–*neighborhood* of *o* in *S* is the same as DBSCAN, but projected in *S* subspace as follows:




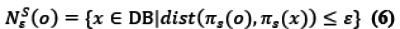




The core object is defined as follows:




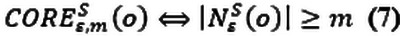




The algorithm begins by generating all one-dimensional clusters using the DBSCAN algorithm. For each detected cluster, it checks whether the cluster also exists in higher dimensions or not. For each k-dimensional subspace *S* ∈ *S_k_*, the algorithm searches all other k-dimensional subspaces *T* ∈ *S_k_* having (k-1) attributes in common and combines them to generate (k + 1)-dimensional candidate subspaces. Based on prior studies [21], we chose the *Midpts* to be in the range from 8 to 128 (with five steps) and the *ε*–*neighborhood* to be from 0.01 to 0.25 (with nine steps). For this study, the initial *Midpts* value was set to 8 and increased by 30 after each run until it reached 128. The *ε*–*neighborhood* value was initially set to 0.01 and was increased by 0.03 until a maximum of 0.25.

#### Cell-Based Approach

Cell-based clustering is centered on cell estimate of the data space. The width of the cells is parametrized by *w*. A cluster *R* contains a set of cells, and each cell contains at least *τ* number of data points. One of the popular cell-based methods is the *MineClus* algorithm, which describes each of these cells as the objects of the cluster by a hypercube with width *w*. These hypercubes are arbitrarily positioned to define a region with frequent data patterns.

A cell-based subspace cluster (*O*, *S*) is defined with respect to the minimum number of objectives τ in cells *CS* of *w* width specified by intervals *I_i_* per dimension *∀i* ∈ *S*. Each interval is part of the common domain *I_i_* = [*l_i_*…*u_i_*] ⊆ [0…*v*] with lower and upper bounds *l_i_* and *u_i_*. For all irrelevant dimensions *∀j* ∈ *D*\*S*, the interval is the full domain *I_j_* = [0…*v*], and the cluster objects *O* = {*o*|*o* ∈ *DB* ∩ *CS*} fulfill |O| ≥ τ [21].

#### Clustering-Oriented Approach

Clustering-oriented approaches focus on the clustering result *R* by specifying objective functions. PROCLUS [22], one of the first top-down subspace clustering algorithms, forms the clusters first and iteratively improves the clustering model. In the PROCLUS algorithm, the number of clusters and the average dimensionality are used as parameters, and data are partitioned into *k* clusters with the average dimension being *l*. A clustering-oriented approach is defined with respect to objective function *f*(*R*), which is based on the entire clustering result *R*, and an optimal value parameter *optF* is a result set *R* with *f*(*R*) = *optF*.

In this case study, we adapted the aforementioned subspace clustering techniques to analyze the PROTECT III data set. Analyses were performed using OpenSubspace [21,23], an open-source framework that extends the WEKA platform [24,25]. All laboratory values were normalized to a scale between 0 and 10 before applying the algorithms.

### Evaluation

Evaluation of unsupervised learning methods, such as cluster analysis, is typically informed by domain expertise. For this work, two clinicians (coauthors of this work [BF and JR]) independently evaluated the results and validated the clusters based on their experiences in the clinical management of TBI as well as clinical research in neurotrauma. The informatician on the team (VS) coordinated the clinician validation process. Mechanistic interpretations for potential markers or associations indicated by clusters were offered based on clinical expertise. To demonstrate alignment of subclustering solutions to a clinical outcome, mortality at 6 months after injury was examined.

Additional evaluation metrics used in this study included F1 score, entropy, coverage, average dimension, and accuracy of classification. The F1 value, a common metric for evaluating clustering algorithms, is defined as the harmonic mean of precision and recall. Entropy is a metric that accounts for clarity of clustering [26]. Coverage characterizes how clusters cover the input data space. Average dimension is the average of number of dimensions that the clusters cover in each run. Accuracy of classification compares the patterns detected in the model in relation to labeled data, such as outcome. Here, the mortality status of TBI patients was used as the outcome. Finally, the performance of subspace clustering algorithms was compared to traditional k-means clustering, which partitions *n* data points into *k* clusters, placing each observation in one of the clusters with neared mean representation. While k-means rely on distance metrics and proximity of observations within individual clusters, subspace methods group data points based on their lower-dimensional subspaces. Given these distinct algorithmic differences between subspace and k-means clustering in formulation of the clustering problem, a direct comparison of the clusters formed and interpretation of clusters may not be appropriate. Instead, we report performance metrics for comparison purposes.

## Results

### Subject Characteristics

Of the 882 study subjects in the parent PROTECT III trial, 643 subjects met the inclusion criteria for this study. Table 1 shows the characteristics of these study subjects at baseline. Ten different laboratory results were used in this study, including blood serum chemistry and hematology results at baseline (Table 2). Coagulation tests, such as the international normalized ratio (INR) and activated partial thromboplastin time, were also included.

**Table 1:** Patient characteristics.

Characteristic		Value (N=643)
Age (years), mean (range)		34 (17-93)
Male sex, n (%)		475 (73.9)
Black people, n (%)		105 (16.3)
Hispanic people, n (%)		97 (15.1)
**Cause of injury, n (%)**		
	Motor vehicle accident	242 (37.7)
	Motorcycle or scooter accident	121 (18.8)
	Pedestrian struck by a moving vehicle	78 (12.1)
	Other	202 (31.4)

**Table 2:** Laboratory results.

Laboratory parameter	Value, mean (range)
Glucose, mg/dL	151.6 (68-554)
Creatinine, mg/dL	1.015 (0.3-4.2)
Potassium, mmol/L	3.667 (1.5-5.8)
Sodium, mmol/L	139.8 (125-157)
Chloride, mmol/L	105.4 (88-130)
Bicarbonate, mmol/L	22.77 (8.0-34.0)
Hemoglobin, g/dL	13.66 (4.9-18.6)
Hematocrit, %	40.31 (14.6-54.2)
Total white blood cell count, ×10^9^/L	14.85 (3.2-41.40)
Platelet count, ×10^3^/mm^3^	249.7 (51-700)

### Application of Subspace Clustering Algorithms to PROTECT III Data

All three types of subspace clustering algorithms (density-based [SUBCLU], cell-based [MineClus], and clustering-oriented [PROCLUS] algorithms) were applied to the PROTECT III data set. The INR, which characterizes the clotting tendency of blood, was identified as one of the distinct clusters. This could represent coagulopathy, a marker of secondary insult in TBI patients [27]. For example, coagulopathy is associated with increased risk of ongoing bleeding and expansion of any intracranial traumatic hemorrhage. One of the clinicians also noted that progressive coagulopathy, which is resistant to correction, is further associated with worse outcomes in TBI patients.

**Figure 1:**
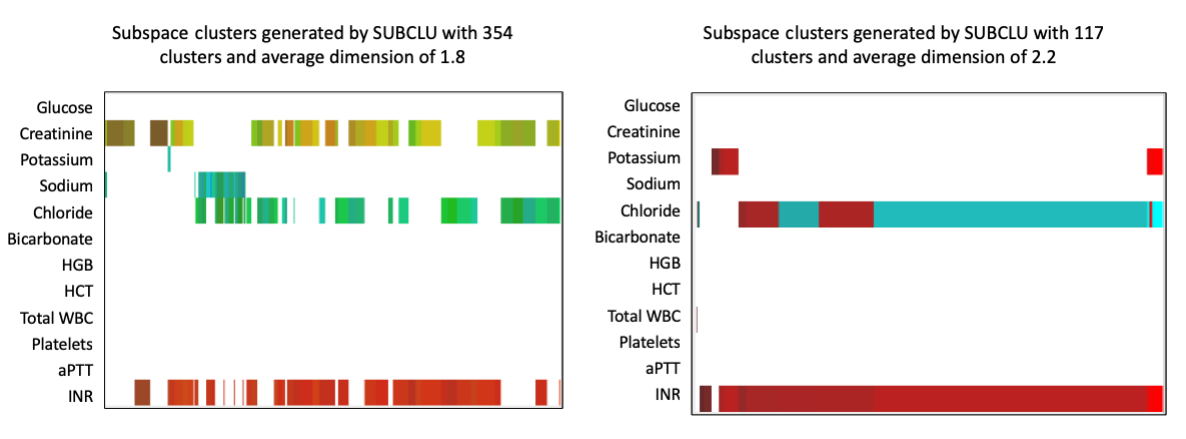


**Figure 2:**
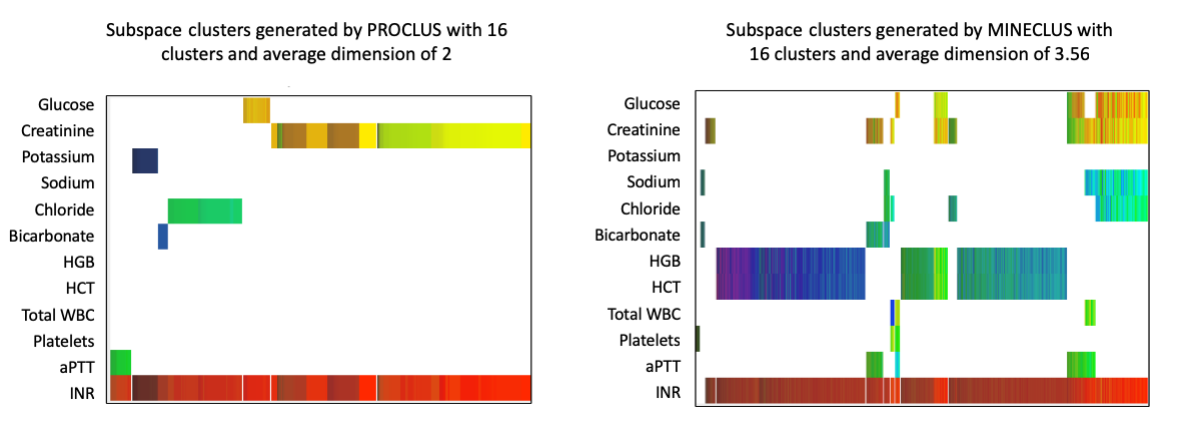


The clustering models also showed a strong relation among INR, chloride, and creatinine. Both clinicians noted and agreed that elevations in chloride levels are often related to fluid administration for treatment of intracranial hypertension or a shock (hypoperfusion) state. Therefore, elevations in these parameters may also be indicators that the clinical team needed to treat a sicker patient more aggressively. Creatinine may be elevated at baseline in patients with chronic illness or may indicate that secondary kidney failure may impact outcome as a complication of TBI. One of the clinicians noted that there is a further relationship between elevated chloride and subsequent elevation in creatinine, though a wide variety of insults may lead to elevations in creatinine. Finally, in models with higher dimensions, a relationship between the hemoglobin level and hematocrit percentage was noted. This relationship is quite intuitive, given that both measure similar properties. These observations are demonstrated in Figure 1 and Figure 2.

The performances of different subspace clustering methods as well as the traditional k-means algorithm on the PROTECT III data set were compared using various evaluation metrics and the mortality status as the outcome (Table 3). The density-based algorithm (SUBCLU) had higher F1 and coverage. The cell-based algorithm (MineClus) had good performance on the F1 measure while having lower number of clusters. The clustering-oriented algorithm (PROCLUS) performed reasonably in terms of accuracy and entropy, while it had the lowest F1 compared to other models. K-means, given its simplicity, was the fastest algorithm, but performed worst in all other metrics.

**Table 3:** Comparison of subspace clustering algorithms.

Evaluation metric	Density-based algorithm (SUBCLU^a^), min-max	Cell-based algorithm (MineClus), min-max	Clustering-oriented algorithm (PROCLUS), min-max	K-means, min-max
F1	0.45-0.69	0.42-0.64	0.36-0.44	0.21-0.30
Entropy	0.45-0.59	0.44-0.55	0.48-0.63	0.51-0.53
Coverage	0.9-1	0.78-0.97	0.43-0.82	0.11-1
Number of clusters	6-1024	6-64	8-32	2-32
Average dimensions	2.3-9	3.2-6.1	2-9	12
Accuracy (%)	81-88	88-88	88-88	51-63
Runtime (s)	367-745,785	58-194	155-402	0.07

^a^SUBCLU: density-connected subspace clustering.

## Discussion

Currently, clinical data used to predict outcomes after TBI come from modeling and validation performed across two older clinical studies in TBI encompassing more than 15,000 patients [28,29]. The covariates that were significant in these prior regression models included glucose and hemoglobin, in addition to clinical predictors such as age and clinical examination. However, the area under the curve of these models is suboptimal. Clusters of data may also incorporate clinical knowledge such as the observation that the combination of lactic acidosis, hypothermia, and coagulopathy at presentation after major trauma imparts poor prognosis. Furthermore, many of these patients do not survive the 72 hours required for inclusion in the current analysis.

Lack of access to multiple data sources has limited further external validation of the proposed methods. Nonetheless, clinician validation is important to inform analyses of data from ongoing observational studies and provide valuable insights into the development of clinically relevant tools for TBI management. This case study serves as a demonstration for such applications. As a next step, focus on temporal data and methods for time-series analyses are warranted.

### Conclusion

This study explored the application and feasibility of subspace clustering techniques for a specific clinical condition, TBI, using clinical data from a randomized clinical trial. The analyses showed the following three clusters of laboratory physiological data: (1) INR, (2) INR, chloride, and creatinine, and (3) hemoglobin and hematocrit. While all subclustering algorithms had a reasonable accuracy in classifying patients by mortality status, the density-based algorithm had a higher F1 score and coverage. Clustering approaches serve as an important step for phenotype definition and validation in clinical domains, such as TBI, where patient and injury heterogeneity are among the major reasons for failure of clinical trials. Results from this study also provide a foundation to develop scalable clustering algorithms for further research and validation.

## Abbreviations

DBSCAN: density-based spatial clustering of applications with noise
INR: international normalized ratio
PROTECT III: Progesterone for Traumatic Brain Injury, Experimental Clinical Treatment–Phase III
SUBCLU: density-connected subspace clustering
TBI: traumatic brain injury
